# Integrating 3D-printed sclero-corneal models into Rigid Gas Permeable contact lens training: A pilot study

**DOI:** 10.1371/journal.pone.0357490

**Published:** 2026-09-09

**Authors:** Nerea Tolón Zardoya, Diana Gargallo Yebra, Jorge Ares García, Francisco J. Ávila Gómez, M. Carmen Lopez de la Fuente, Francisco J. Segura Calvo, M. Concepción Marcellan Vidosa

**Affiliations:** Department of Applied physics, University of Zaragoza, Zaragoza, Spain; The Ohio State University, UNITED STATES OF AMERICA

## Abstract

**Purpose:**

Rigid gas permeable contact lenses provide excellent optical quality and oxygen permeability, but their fitting process is complex. Traditional teaching methods, such as student-to-student practice, present limitations regarding safety and corneal geometry diversity. A kit of 3D-printed Sclero-Corneal Surfaces (SCSs) has been proposed as a hybrid tool combining hands-on lens manipulation with the flexibility and geometry diversity of simulations. This study evaluated the viability of these artificial SCSs for rigid gas permeable contact lenses training and used questionnaires to assess students’ perceptions, as well as learning gains in fluorescein pattern interpretation.

**Methods:**

Optometry students were assigned to either a control group or an experimental group. Both groups first practiced corneal contact lens fittings on peers and completed two questionnaires: one evaluating their initial expectations and concerns related to the fittings, and a second assessing their baseline knowledge level. The experimental group then performed fittings using only the artificial models and completed an additional questionnaire examining perceived pedagogical benefits. Finally, all students repeated the knowledge questionnaire. Responses were analyzed using McNemar tests (p < 0.05) to evaluate changes in perceptions, confidence, and knowledge before and after the training.

**Results:**

After real eye practice, the experimental group perceived fluorescein interpretation and lens adjustment as the most challenging tasks, while ocular discomfort was cited as a demotivating factor. After the SCSs session, over 90% of the experimental group found the new method realistic, more than 80% valued the absence of patient discomfort, and 100% of students highlighted the improved understanding of fittings dynamics. Although the knowledge questionnaire showed no statistically significant differences between groups, both reached comparable levels of proficiency.

**Conclusions:**

Artificial SCSs provide a safe, engaging, and complementary approach to traditional RGP-CL training, enabling more fittings in less time without ocular risk and effectively bridging theoretical knowledge with clinical practice.

## Introduction

Rigid gas permeable contact lenses (RGP-CLs) offer excellent oxygen permeability [1] and optical quality, making their fitting a key clinical skill for contact lens (CL) practitioners [2]. Although RGP-CLs account for approximately 15% of CL fittings worldwide [3], corneal RGP-CLs are the preferred option for managing patients with irregular corneas, including keratoconus and other corneal ectasias [4]. Rigid lens materials are also widely used for myopia control with orthokeratology [5]. However, corneal RGP-CL fitting is considerably more complex than soft CL fitting [6], requiring accurate interpretation of fluorescein patterns together with an understanding of corneal geometry and lens dynamics [7]. Consequently, developing these competencies is essential but remains a challenging component of CL training [8–10].

Corneal RGP-CL fitting is an iterative clinical process in which practitioners sequentially assess different trial lenses. For each lens, the post-lens tear film fluorescein pattern, centration relative to the pupil, movement, lid interaction, tear exchange, visual performance, and patient comfort are evaluated to determine whether further modifications to the lens parameters are required [2,7]. Mastering these clinical skills is therefore an essential component of CL education [9].

Traditional teaching methods typically involve student-to-student practice, with one student acting as the practitioner and the other as the patient, alternating roles throughout the session. This approach provides hands-on experience in lens insertion, evaluation, and removal while promoting active learning and communication skills. However, exposure to different fitting scenarios is naturally limited by the range of corneal geometries represented within a student cohort, and students have relatively few opportunities to repeatedly observe and compare different fluorescein patterns before applying these concepts in real-eye fitting.

In addition, repeated handling of corneal RGP-CLs requires rigorous disinfection between uses and may cause ocular discomfort during repeated peer fitting. Although, to our knowledge, no published studies have specifically documented infectious complications or other adverse events during corneal RGP-CL training, microbial contamination associated with RGP-CL wear has been reported in the contact lens literature [11,12]. Furthermore, conventional peer-to-peer practice provides only limited opportunities to observe the wide range of fluorescein fitting patterns encountered in clinical practice. These educational and practical considerations support the incorporation of complementary teaching strategies that provide a standardized and efficient learning environment while preserving the educational value of conventional clinical training.

Alternatively, instructors can utilize software simulators [9,13] to teach the fitting process. Unlike training with real eyes, this approach eliminates the logistical and safety issues related to lens disinfection. This facilitates the study of how each adjustment affects lens fitting in a precise and efficient manner. However, this methodology lacks the tactile and manual components of clinical training and does not reproduce dynamic lens behavior, which are important aspects for developing full clinical competence [7,14,15].

To bridge this gap, we explored the use of a kit of artificial Sclero-Corneal Surfaces (SCSs), consisting of 3D-printed models reproducing different scleral and corneal geometries for corneal RGP-CL fitting practice. Students place trial corneal RGP-CLs directly onto these models to observe fluorescein patterns under different fitting conditions. This method combines the hands-on advantages of real lens manipulation with the controlled variability of simulation-based learning, allowing students to practice different fitting conditions safely, without requiring direct ocular contact.

This study therefore aimed to evaluate the use of a 3D-printed SCS kit as a complementary resource for teaching corneal RGP-CLs fitting. Beyond implementing this new training method, it was crucial to explore how students perceive both traditional and alternative practice approaches. Understanding their opinions, challenges, and sources of apprehension can provide valuable insights into the educational feasibility and acceptance of using SCSs. To achieve this, pre- and post-training questionnaires were administered to assess students’ attitudes toward conventional student-to-student practice, evaluate their satisfaction with the new SCS-based methodology, and measure gains in confidence and fluorescein pattern interpretation.

## Materials and methods

### Participants

A quasi-experimental design was implemented. Students enrolled in the course during the third year of Optics and Optometry at a higher education institution participated in the study. Students had previously been allocated to two practical laboratory groups according to the standard course organization. These groups were then randomly assigned to either the Control Group (C.G.) or the Experimental Group (E.G.). As all participants were enrolled in the same academic year and had completed the same coursework before the study, they were considered to have an equivalent level of prior training and experience. The E.G. included 18 students (77.8% female and 22.2% male; mean age 20.7 ± 1.4 years) and the C.G. included 19 students (57.9% female and 42.1% male; mean age 20.8 ± 1.1 years). Participants were prospectively recruited during September 2024, and the study was conducted from September to November 2024. All participants provided written informed consent prior to their inclusion.

### Materials

**Fig 1:**
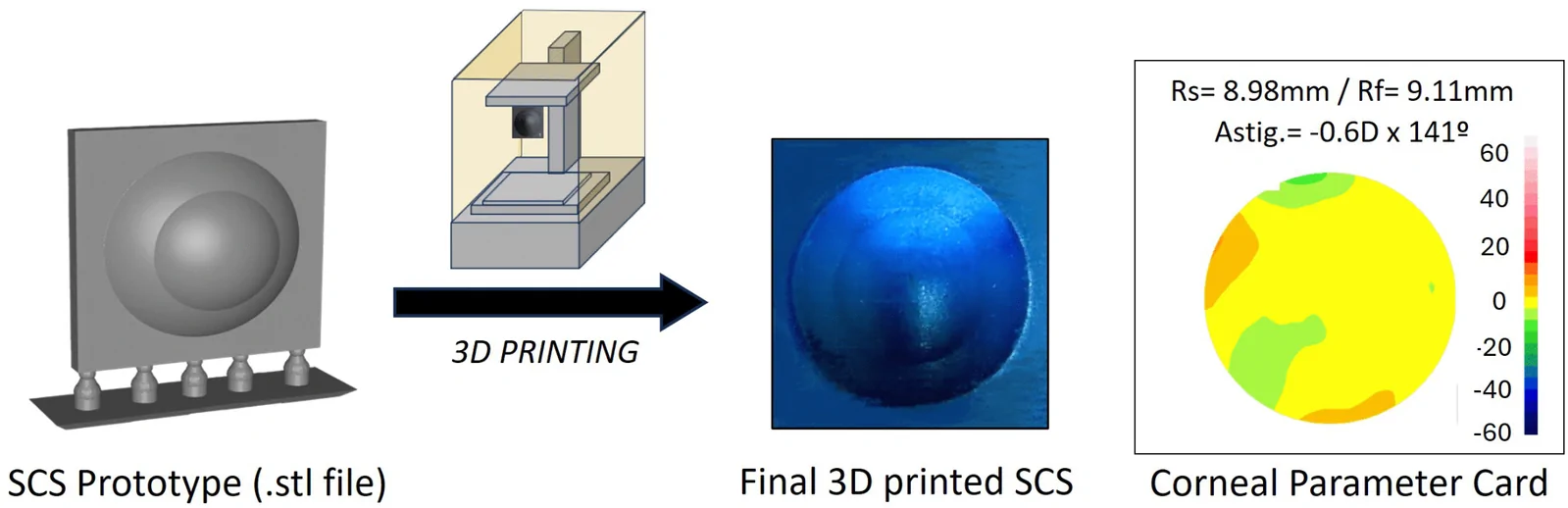
3D Printing process. Left: Stereolithography file for 3D printing. Center: 3D-printed SCS. Right: Corneal parameter card. Legend: Rs: Steep radii, Rf: Flat radii, Astig.: astigmatism.

The SCS kit comprised 23 3D-printed models, each consisting of a scleral base with a customized corneal surface representing different corneal geometries, mounted on a 24 × 24 mm square base (Fig 1) [16]. The SCSs were digitally designed and fabricated using a Peopoly MOAI 130 stereolithography 3D printer (Peopoly, China, 2018), which employs UV-laser scanning to selectively polymerize acrylic resin [17]. To ensure anatomical accuracy, each surface was measured with the Eye Surface Profiler (Eaglet-Eye B.V., The Netherlands) [18], a profilometry-based sclero-corneal topographer [19]. These measurements informed the creation of the Corneal Parameter Card (Fig 1), which summarizes the primary geometric features (Rs, Rf, and astigmatism) of each model.

Training sessions were conducted in Optometry laboratories fully equipped with a comprehensive range of spherical corneal RGP-CLs, Burton lamps, absorbent paper, and pipettes for fluorescein instillation. Commercially available spherical corneal RGP-CLs were utilized for all fitting simulations, both on real eyes and SCS models. Corneal RGP-CLs covered a range of base curve radii from 7.00 to 8.80 mm and total diameters from 8.8 to 10.0 mm, allowing students to evaluate different fitting scenarios by modifying these parameters.

### Procedure

#### First session.

Prior to the study, students had received a traditional lecture with whiteboard and slide presentation on RGP-CL design and fluorescein pattern interpretation, covering the basic principles of lens fitting, including the evaluation of the central, mid-peripheral, and border fitting zones through fluorescein pattern interpretation. Students also learned the theoretical implications of modifying lens parameters, including base curve radius, overall diameter, optic zone diameter, and peripheral curve design, on the resulting fluorescein pattern.

This practical session represented the participants’ first hands-on experience with corneal RGP-CL fitting. Both groups participated in a 150-minute traditional student-to-student training session in which students worked in pairs or triads, alternating between practitioner and patient roles. The student acting as the practitioner selected and fitted a corneal RGP-CL on the classmate assigned as the patient. After inserting the lens, the practitioner assessed the fluorescein pattern, lens movement and dynamic behavior, measured visual acuity, performed the corresponding over-refraction and removed the CL. Based on these findings, the student then decided whether any modification of lens parameters was required and, when appropriate, repeated the fitting to achieve an optimal outcome. Throughout the session, an instructor supervised the activity and provided guidance when necessary.

Each group completed approximately 2–3 full fittings, ensuring that all participants practiced both the clinician and patient roles. Activities included hands-on practice of insertion and removal techniques, systematic evaluation of the fitting outcomes, and decision-making regarding lens adjustments under instructor supervision. Following this first session, all students completed two questionnaires: the “Real eyes Opinion Questionnaire” and the baseline “Knowledge Questionnaire”, described in detail in Section 2.5.

#### Second session.

The C.G. repeated the traditional procedure, performing two or three fittings. In contrast, the E.G. completed a 90-minute SCS-based session structured as a collaborative workshop, during this session, a peer-learning discussion was organized where students worked in pairs (utilizing the portable SCS kit and Burton lamps to evaluate fluorescein patterns simultaneously) to complete the following steps:

1. Instructor explanation of SCS fabrication and topographic map scale interpretation (Fig 1).
2. Instructor-led fitting of spherical corneal RGP-CLs on spherical SCSs using a saline-fluorescein mixture (ensuring a bubble-free interface and removing excess fluid).
3. Discussion of three spherical fitting scenarios to practice pattern identification and parameter optimization. “What type of fitting does this fluorescein pattern represent?” and “What adjustments would you make to the CL parameters to optimize the fitting?.”
4. Using spherical SCSs, students identified each fluorescein pattern as steep, aligned, or flat and selected a different trial lens by modifying only the base curve radius and overall diameter. Adjustments were made according to the observed fluorescein pattern (steeper for flat fittings and flatter for steep fittings), without predefined parameter increments, while the instructor confirmed the students’ interpretation and rationale for the selected parameter changes. The objective of this exercise was to develop students’ understanding of the relationship between fluorescein patterns and lens parameter modifications.
5. The instructor demonstrated three practical cases on toric SCSs with spherical CLs, emphasizing how to interpret topographical maps in the presence of corneal astigmatism. This exercise was not intended to suggest spherical CLs as the standard option for toric corneas, but rather to help students visualize the distribution of the tear film under the lens and to practice the description of the fluorescein pattern with the help of the elevation topographic map.
6. Students applied the previous methodology to toric SCSs to refine their fluorescein pattern description and interpretation skills.

**Fig 2:**
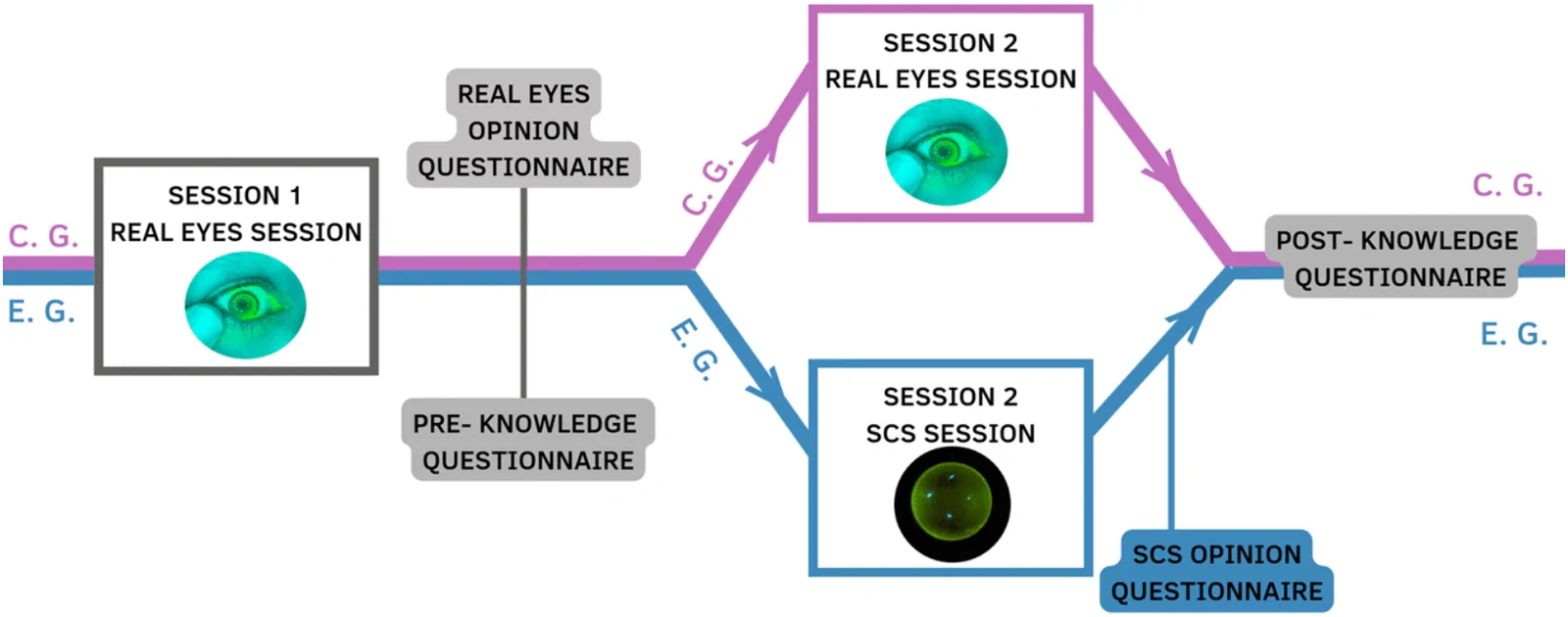
Timeline diagram illustrating the study process. The activities conducted by each group, and the questionnaires. Legend: Both groups marked on the central line, Control group: Upper section in purple and Experimental group: Lower section in blue.

After this session, the E.G. completed the “SCS Opinion Questionnaire”. Both groups repeated the “Knowledge Questionnaire”. Fig 2 provides a schematic overview of the sequence of activities conducted throughout the study, illustrating the different stages completed by each group. Questionnaires are described in detail in Section 2.5.

### Questionnaires

Three ad hoc instruments were developed to assess the study’s learning objectives:

1. **Real eyes Opinion Questionnaire** (see S2): designed to explore students’ fears, concerns, overall students’ confidence and the most-time consuming stages of the traditional practice. In addition, the perceived complexity of each fitting stage and identified the aspects that most discouraged students during traditional practice.
2. **SCS Opinion Questionnaire** (see S3), designed to assess students’ perceptions and improvements of the new SCS training method. Exploring the perceived usefulness for skill development, efficiency without lens disinfection, the educational value of the corneal topography maps and preferred method for practicing different RGP-CLs fitting stages (Real eyes, SCSs or a combination).
3. **Knowledge Questionnaire Pre-Post** (see S4), a pre-post multiple-choice test featuring clinical images of fluorescein patterns on real eyes to measure objective learning gains.

### Data analysis

Student perceptions were analyzed as frequency percentages. For the Knowledge Questionnaire, performance was evaluated using the percentage of correct answers with 95% confidence intervals (CIs), calculated via the Agresti and Caffo method [20] (n (C.G)=19; n (E.G) =18).

To detect significant shifts within and between groups, the McNemar test was employed [21,22], as it is uniquely suited for paired nominal data. Statistical processing was executed in Python (Google Colab) using statsmodels and NumPy, while graphical representations were generated in Microsoft Excel.

## Results

The following results are organized according to the different phases of the intervention. First, the outcomes from both groups corresponding to the Opinion Questionnaire after real eyes session are presented, followed by the results of the E.G. regarding the Opinion Questionnaire after SCS session. Finally, results obtained from the Knowledge Questionnaire for both groups are reported.

### Real eye opinion questionnaire

A minority of students reported spending significant amount of time on cleaning and disinfecting the CLs (38.9% E.G., 21.1% C.G.). In contrast, 55.6% of the E.G. and 36.8% of the C.G. indicated that they spent a considerable amount of time on the insertion and removal steps. Regarding interruptions to ask the instructor for assistance, 72.2% of the E.G. and 66.7% of the C.G. responded “sometimes”. When asked about the importance of working in a group to discuss lens fittings, 100.0% of the E.G. and 89.5% of the C.G. considered it essential. Results are presented in Table 1.

**Table 1:** Results from the Opinion Questionnaire after Real eyes session (percentages of students).

*Question*	*Responses*	*E.G. 18 st. (%)*	*C.G. 19 st. (%)*
**Time spent on cleaning and disinfecting**	*Very little*	0.0	0.0
	*Little*	5.6	21.1
	*Average*	**55.6**	**47.4**
	*A lot*	38.9	21.1
	*Too much*	0.0	10.5
**Time spent on insertion/removal**	*Very little*	0.0	0.0
	*Little*	5.6	5.3
	*Average*	38.9	**52.6**
	*A lot*	**55.6**	36.8
	*Too much*	0.0	0.0
**Interruptions to ask the instructor for assistance**	*No*	27.8	27.8
	*Sometimes*	**72.2**	**66.7**
	*Yes*	0.0	5.6
**Importance of discussion in group**	*Not necessary*	0.0	10.5
	*Necessary*	**100.0**	**89.5**

Marked in bold the majority of responses, st.: students.

**Fig 3:**
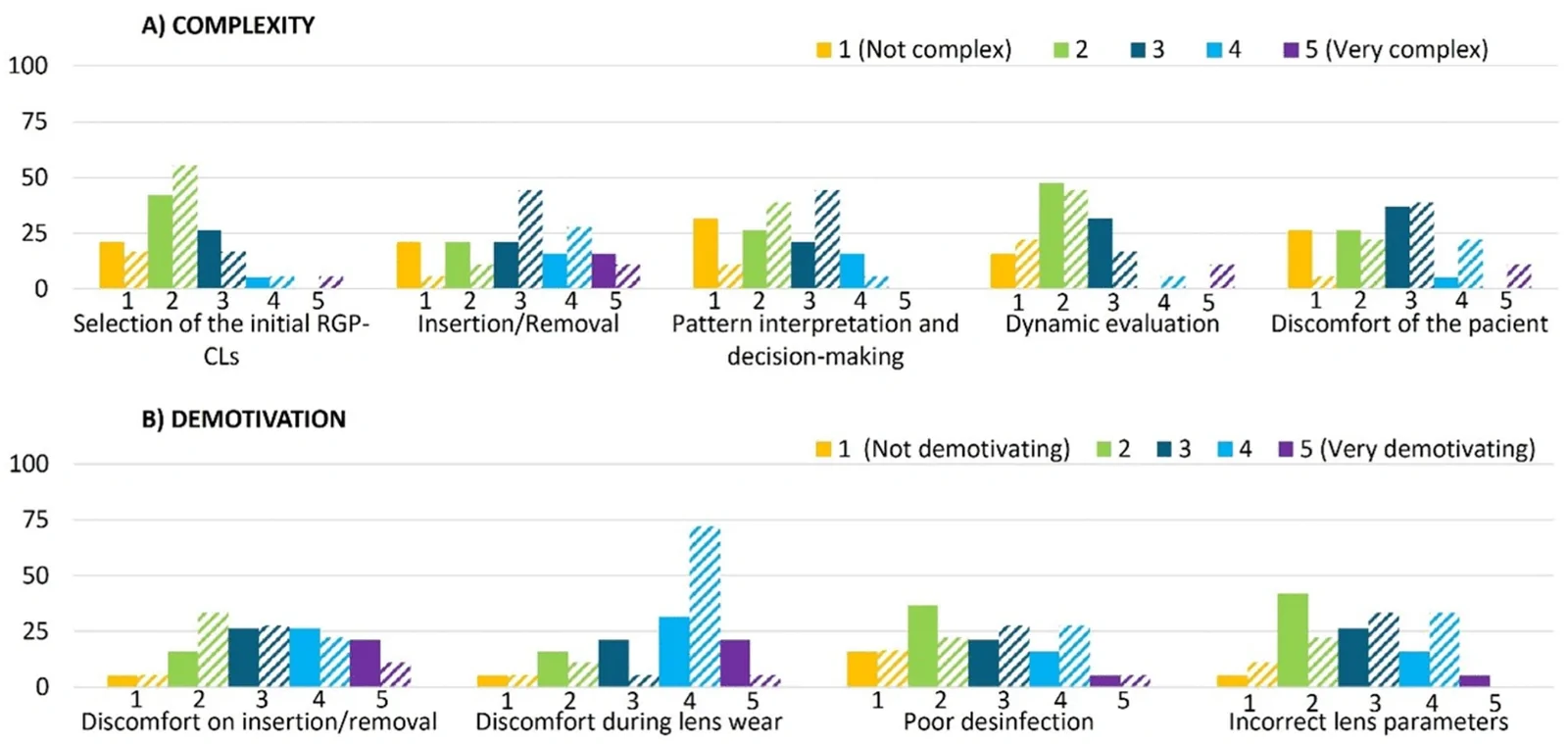
Results for complexity of RGP-CL fitting stages (A) and demotivation of RGP-CL fitting aspects (B).

Fig 3 presents the results for two rating-scale items (1: low to 5: high). Regarding task complexity (Fig 3 A), both groups rated lens selection and dynamic evaluation as relatively simple (level: 2). However, the E.G. found insertion/removal moderately complex (level: 3; 44.4%), while the C.G. rated it lower (level: 2; 44.4%). Interestingly, fluorescein interpretation and lens adjustment were perceived as less complex by both groups (38.9% of E.G. level: 2; 31.6% of C.G. level: 1). Managing patient discomfort during evaluation was consistently rated as complex (level: 3) by both cohorts.

Regarding demotivating factors (Fig 3 B), lens wear discomfort emerged as the primary deterrent, particularly for the E.G. (level: 4; 72.2% vs. C.G. 31.6%). In contrast, discomfort during insertion/removal was less demotivating (33.3% of E.G. level: 2; 26.3% of C.G. level: 3). Other factors, such as poor disinfection and incorrect lens parameters, received moderate-to-low demotivation scores (levels ranging from 2 to 4), with slight variations between groups.

### SCS opinion questionnaire

After the SCS session, most students rated the experience as realistic and pedagogically valuable as shown in Table 2. Over 90% found the SCSs similar to the human eye and 72.2% valued the fluorescein pattern simulation as highly useful, and most students (61.1%, Table 2) reported performing more fittings in less time compared with real eye practice. The absence of discomfort, cited by over 80% of participants, and the group discussions, highlighted by over 90%, were identified as key factors enhancing learning. All students considered the use of topographic elevation maps helpful for practicing RGP-CL fittings, and all agreed that 3D printing technology added appeal to the activity, and 94.4% felt it improved the manipulation of RGP-CLs (Table 2).

**Table 2:** Results from the Opinion Questionnaire after SCS session (percentages of students).

*Question*	*Responses*	*E.G. 18 st. (%)*
**Similarities between SCSs and real eyes**	None	0.0
	Slight similarity	5.6
	Very similar	**94.4**
**Utility of simulating fluorescein patterns on SCSs**	(Not useful at all) 1	0.0
	2	0.0
	3	0.0
	4	27.8
	(Very useful) 5	**72.2**
**Have you acquired knowledge or skills you can apply in your future professional practice?**	*Very little*	0.0
	*Little*	0.0
	*Average*	11.1
	*A lot*	**77.8**
	*Very much*	11.1
**More nº fittings on SCSs or on real eyes**	*More on SCSs*	**61.1**
	*More on real eyes*	16.7
	*Same*	22.2
**Did you learn more by not having to worry about discomfort?**	*No*	16.7
	*Yes*	**83.3**
**Did the group discussion help you?**	*No*	5.6
	*Yes*	**94.4**
**Time was efficiently used as proper disinfection was not required?**	*No*	0.0
	*Yes*	**100**
**Learning experience more dynamic and engaging**	*No*	11.1
	*Yes*	**88.9**
**3D Technology adds appeal to the sessions?**	*No*	0.0
	*Yes*	**100**
**The topographic map improved the learning process**	*No*	0.0
	*Yes*	**100**
**Use of SCSs helped improve manipulating CLs?**	*No*	5.6
	*Yes*	**94.4**
**Utility of highly astigmatic corneal surfaces**	*No*	5.6
	*Yes*	**94.4**
**Enough time used on SCSs?**	*No, too much time*	5.6
	*No, too little time*	5.6
	*Yes, it was appropriate*	**88.9**
**Timing more useful to use SCSs?**	*At the beginning*	33.3
	*At the time as real eyes*	**55.6**
	*After real eyes*	11.1
**The beneficial use of SCSs in future contactology training**	*No, no it isn’t useful*	0.0
	*Yes, it will be useful*	**100**
**SCSs improved the understanding of RGP-CLs fittings?**	*No*	0.0
	*Yes*	**100**

Marked in bold the majority of responses. st.: students.

This training session would likely be more effective if conducted simultaneously with real eye practice, as reported by 55.6% of the students. Moreover, all participants indicated that the session improved their understanding of RGP-CL fitting (Table 2).

**Fig 4:**
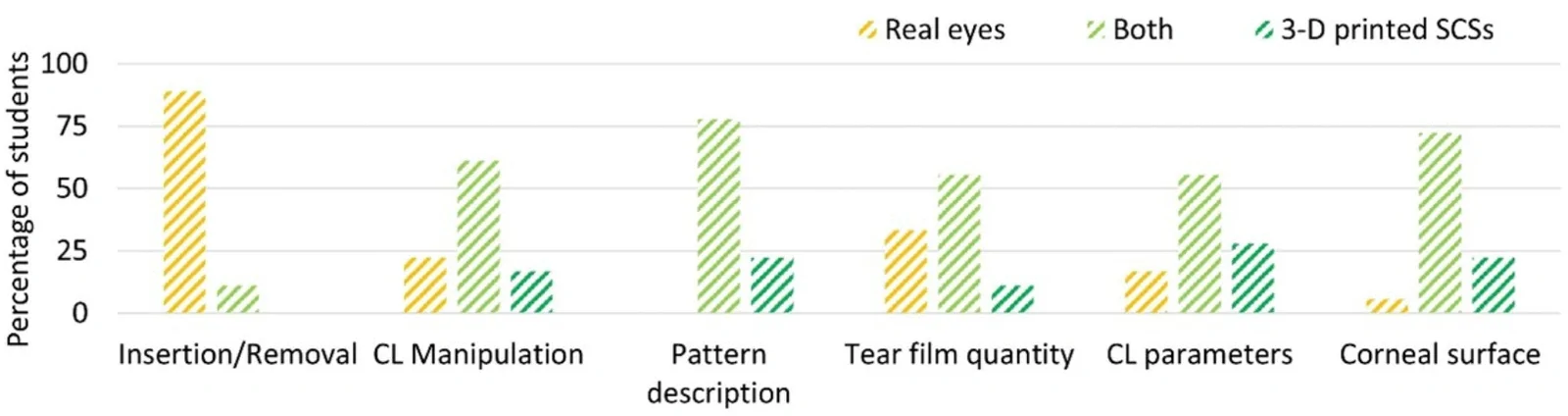
Student percentages. Percentages for the most suitable method for each component of the RGP-CLs fitting process selected by students.

In Fig 4 results for one item are shown, in which students were asked which method (real eyes, 3D-printed SCSs, or both) they considered most appropriate for different phases of the RGP-CL fitting process (insertion/removal, lens manipulation, fluorescein pattern description, tear film quantity, parameter adjustments, and knowledge of corneal geometry) (Fig 4). Real eyes were mainly preferred for insertion and removal (88.9%), for all other steps, most students reported that both methods were useful: lens manipulation (61.1%), fluorescein pattern description (77.8%), tear film quantity (55.6%), parameter adjustments (55.6%), and corneal geometry knowledge (72.2%). Notably, no students selected only real eyes for fluorescein pattern interpretation, highlighting the value of SCSs in training (Fig 4).

### Knowledge questionnaire

**Fig 5:**
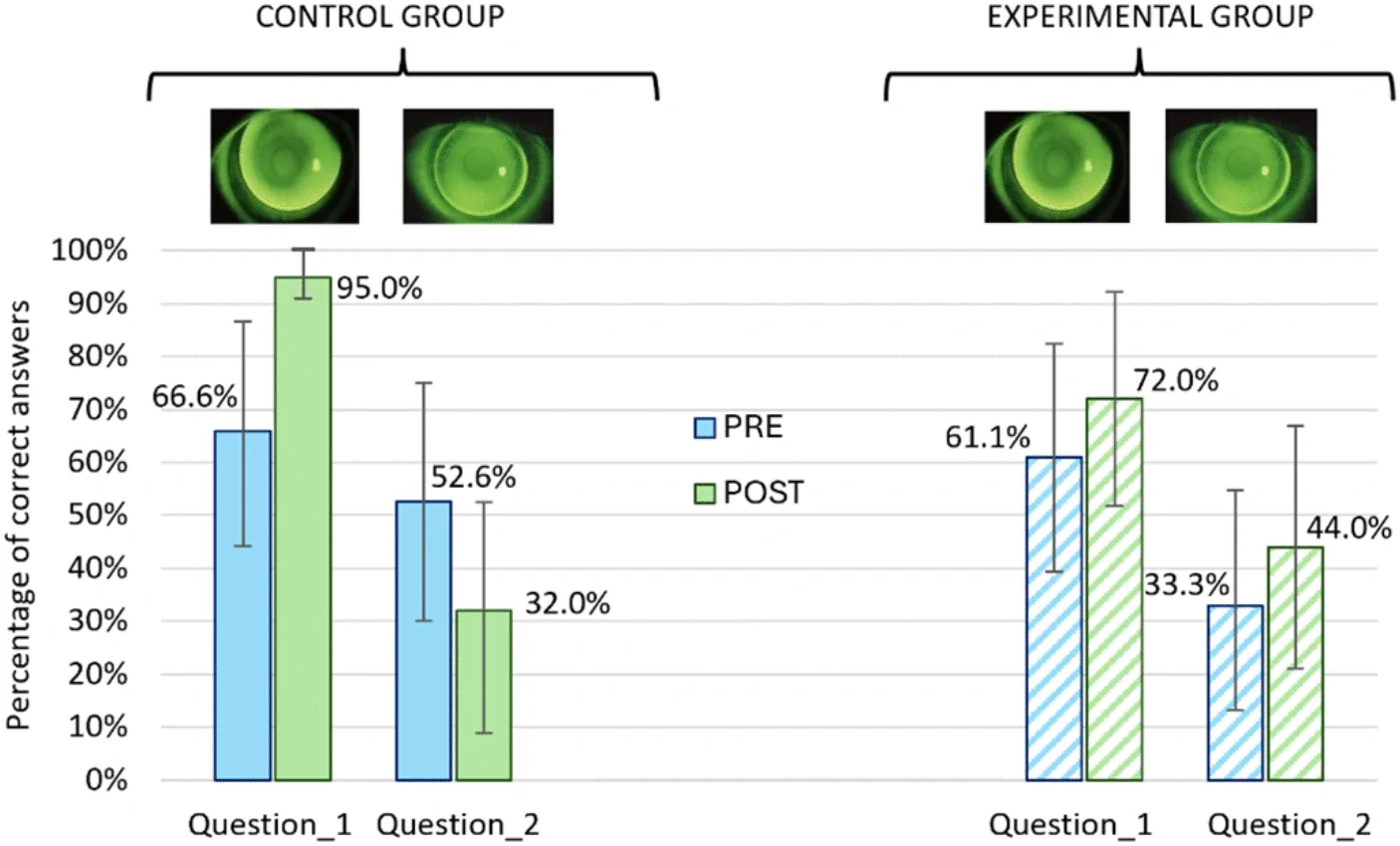
Percentage of correct answers in the knowledge questionnaire pre- and post- intervention (95% CI). Control group: Solid colors (left), Experimental group: striped colors (right).

Fig 5 presents the percentage of correct answers and their respective 95% CIs for both groups. Baseline results are shown in blue (solid for C.G., striped for E.G.), while post-intervention results are displayed in green. The E.G. demonstrated a consistent improvement in both items: correct responses increased from 61.1% to 72.0% for Question 1, and from 33.3% to 44.0% for Question 2. In contrast, the C.G. showed heterogeneous outcomes; although performance in Question 1 improved (66.6% to 95.0%), Question 2 saw a decline (52.6% to 32.0%). Despite these trends, McNemar’s test confirmed that changes within and between groups were not statistically significant (p > 0.05).

## Discussion

The successful fitting of RGP-CLs is a complex clinical task that requires a thorough knowledge of corneal geometry and lens design. Due to the technical demands and precision involved in corneal RGP-CLs fitting, comprehensive and effective training is essential to ensure optimal patient outcomes and clinical confidence among future practitioners. Enhancing educational strategies in this area is therefore critical to better prepare students for real-world clinical challenges and to improve the quality of care in CLs practice.

To address this need, we proposed a new method in which students manipulate RGP-CLs on 3D-printed surfaces representing various corneal geometries. This hands-on approach increases student engagement and provides the capability to fit different CLs, allowing learners to understand how adjustments to lens parameters affect the fitting. It also allows real-time observation of fluorescein patterns, reinforcing core fitting concepts through practical experience.

Our findings align with recent research by Wu et al. [23], who demonstrated that 3D-printed ocular simulators improve student satisfaction and performance by providing a standardized, low-stress environment. While digital platforms, such as the virtual system explored by Li et al. [24], offer valuable cognitive frameworks, they lack the physical interaction inherent to our SCS models, which enable students to develop essential manual skills and confidence prior to clinical application.

Our study implements a tactile, in-person educational model using a kit of SCSs. While ocular simulators and digital platforms support the move away from traditional peer-fitting strategies, our model offers a controlled environment in which students can practice fluorescein pattern interpretation before applying these skills during conventional real eye training. These approaches underscore the value of integrating both virtual and physical simulations in optometric education, each offering distinct pedagogical advantages depending on the learning objectives.

In our experimental process, as students began practicing RGP-CLs fitting on their classmates, they reported challenges such as discomfort during procedures and a substantial time spent on insertion, removal, and lens disinfection (Table 1). These issues motivated the introduction of in-class simulations using the kit of SCSs, designed to replicate corneal geometry and allowing practice of different fitting types.

The strong perceived usefulness of the activity suggests that incorporating RGP-CL fitting on SCSs can effectively enhance students’ learning (Table 2). Reports on improved practical skills, together with the perception of a more dynamic and engaging experience, indicate that combining traditional instruction with SCS-based exercises may promote deeper insight and motivation. Moreover, the positive reception of fluorescein pattern simulations and topographic maps underscores the potential of SCSs to offer meaningful visual and tactile practice that complements standard clinical training (Table 2).

The preference for using both SCSs and real eyes methods across most phases of the fitting process suggests that students recognize the complementary educational value of each method (Table 2). While real eyes offer authentic tactile experience during insertion and removal, SCSs allow repeated practice and visualization of fluorescein patterns, including those associated with highly toric corneal models (Table 2).

The SCS-based method proved particularly useful for visualizing and practicing fluorescein patterns, enabling students to observe approximately twice as many RGP-CL fittings in less time and to explore a wider range of corneal geometries (Table 2). However, this approach was limited to fluorescein pattern evaluation, whereas traditional student-to-student practice also involves assessing lens movement, patient comfort, and insertion and removal techniques.

Therefore, both methods should be considered complementary, as each contributes distinct yet essential aspects to the learning process rather than the proposed approach being viewed as a replacement for conventional student-to-student practice. Although corneal RGP-CLs represent a relatively small proportion of CL fittings, competence in their fitting remains essential because of their importance in specific clinical indications. One potential educational advantage of the proposed approach is that it may facilitate the acquisition of basic corneal RGP-CL fitting skills in less time, which could contribute to a more efficient allocation of curricular time while maintaining appropriate training in corneal RGP-CL fitting and allowing time for training in other CL fitting modalities. Further studies with larger cohorts and objective clinical performance measures are needed to confirm its educational effectiveness.

Furthermore, SCS-based training encouraged collaborative decision-making, eliminated the need for lens disinfection, and reduced students’ fear of causing discomfort to a classmate. The corneal elevation maps associated with each SCS also provided predictive insights into lens behavior, enhancing clinical reasoning and overall knowledge of RGP-CL fitting (Table 2).

Although the E.G. exhibited an increase in their responses to the Knowledge Questionnaire, statistical analysis using the McNemar test did not reveal significant differences. This suggests that, while the SCS training may offer pedagogical value, its short-term impact on specific knowledge acquisition remains inconclusive. The absence of statistically significant improvements highlights the need for more extensive implementation and larger sample sizes to better evaluate the method’s true educational potential.

### Limitations of the study

Despite these positive outcomes, several limitations should be acknowledged. The proposed SCS-based method addresses only one component of the corneal RGP-CL fitting process: the interpretation of fluorescein patterns and the assessment of how modifications to lens parameters affect those patterns. Consequently, it does not replace the acquisition of the remaining clinical skills required for complete corneal RGP-CL fitting during conventional real-eye training. Therefore, this approach should be regarded as an initial step in the acquisition of corneal RGP-CL fitting skills rather than a replacement for traditional clinical training.

The practical session was designed as an initial step in training for corneal RGP-CL fitting, using a Burton lamp before introducing slit-lamp biomicroscopy, the clinical gold standard for fitting assessment [25]. Although slit-lamp enables a more precise evaluation of fluorescein intensity, sub-lens tear film distribution, and the dynamic behaviour of the lens, the use of a Burton lamp allowed students to focus on the interpretation of fluorescein patterns and their relationship with lens parameter modifications without the additional technical demands associated with slit-lamp examination.

Consequently, the educational outcomes reported in this study should be interpreted as reflecting students’ ability to interpret fluorescein patterns and relate them to lens parameter modifications, rather than as an assessment of the clinical competencies required for comprehensive RGP-CL fitting using slit-lamp. In future iterations, incorporating slit-lamp as a second stage of the SCS-based training would expand the learning process by enabling students to assess additional fitting characteristics, including the dynamic behaviour of the lens on vertically mounted SCSs, before progressing to conventional real-eye training.

This was a pilot study with a limited sample size and relatively short (90-minute) sessions, which may explain why the Knowledge Questionnaire did not yield statistically significant differences (p > 0.05). Furthermore, the current models lack anatomical landmarks (such as an iris, pupil, or eyelid margins) which are necessary to accurately determine lens diameter, centration, and natural fluorescein distribution.

Additionally, the study did not evaluate toric lenses on toric surfaces, nor did it assess whether repeated peer-to-peer practice in the Control Group improved confidence, limiting direct comparisons between the two methodologies. Furthermore, while the Knowledge Questionnaire provided valuable insights, its scope was restricted to a specific set of fluorescein patterns.

## Conclusions

The SCS-based training method provides a controlled environment for practicing corneal RGP-CL fitting that facilitates repeated observation of different fluorescein fitting patterns and exploration of lens parameter adjustments across a wider range of corneal geometries, while reducing students’ concerns about discomfort associated with repeated peer fitting. It enhances the knowledge of RGP-CL fitting through a more dynamic and engaging learning experience. Although this approach cannot fully replicate real eye conditions, it enables RGP-CLs manipulation and fluorescein patterns understanding in a realistic context. Therefore, SCSs serve as a valuable complement to traditional student-to-student practice, promoting skill development while reducing discomfort and safety concerns.

## Supporting information

S1 Table Sclero-corneal Surfaces parameters. Geometrical parameters of the SCSs of the kit measured with the Eye Surface Profiler (Legend: R: Radii; Rs: Steep Radii; Rf: Flat Radii; Astigm.: Astigmatism; < Ecc > : Mean eccentricity of both main meridians).
(DOCX)

S1 File Real eyes Opinion questionnaire. Questions of the Questionnaire after Real eyes session.
(DOCX)

S2 File Sclero-corneal surfaces Opinion questionnaire. Questions of the Questionnaire after the session using SCSs.
(DOCX)

S3 File Knowledge questionnaire. Real eyes fluorescein pattern questions of the Knowledge questionnaire.
(DOCX)
