# Supplementary material for: Integrating 3D-printed sclero-corneal models into Rigid Gas Permeable contact lens training: A pilot study
Source: PLoS One. 2026 Sep 9;21(9):e0357490. doi: 10.1371/journal.pone.0357490 (PMC13557353; doi:10.1371/journal.pone.0357490)
Supplement: Supplementary file 1 [file pone.0357490.s001.docx]

**S1 Table. Sclero-corneal Surfaces parameters.** Geometrical parameters of the SCSs of the kit measured with the Eye Surface Profiler (Legend: R: Radii; Rs: Steep Radii; Rf: Flat Radii; Astigm.: Astigmatism; <Ecc>: Mean eccentricity of both main meridians).

| **SPHERIC SCSs** | | **TORIC SCSs** | | | | | | | | | | | |
| --- | --- | --- | --- | --- | --- | --- | --- | --- | --- | --- | --- | --- | --- |
|  |  | Toric Against the rule | | | | Toric with the rule | | | | Oblique toric | | | |
| R (mm) | <Ecc> | Rs (mm) | Rf (mm) | Astig. (D) | <Ecc> | Rs (mm) | Rf (mm) | Astig. (D) | <Ecc> | Rs (mm) | Rf (mm) | Astig. (D) | <Ecc> |
| 7.04 | 0.38 | 6.95 | 7.21 | 1.80 | 0.59 | 6.65 | 7.65 | 6.60 | 0.75 | 7.72 | 7.88 | 0.90 | 0.52 |
| 7.25 | 0.64 | 7.26 | 7.58 | 2.00 | 0.83 | 7.26 | 8.23 | 5.40 | 0.75 |  |  |  |  |
| 8.31 | 0.84 | 7.81 | 8.09 | 1.50 | 0.23 | 7.79 | 8.63 | 4.20 | 0.69 |  |  |  |  |
| 9.05 | 0.45 | 7.87 | 8.05 | 0.90 | 0.48 | 7.90 | 8.09 | 1.00 | 0.59 |  |  |  |  |
| 9.20 | 0.66 | 8.01 | 8.35 | 1.70 | 0.41 | 7.94 | 8.24 | 1.60 | 0.56 |  |  |  |  |
| 9.20 | 0.87 | 8.80 | 9.08 | 1.20 | 0.40 | 8.02 | 8.80 | 3.80 | 0.89 |  |  |  |  |
|  |  | 8.83 | 9.16 | 1.40 | 0.63 | 8.04 | 8.59 | 2.70 | 0.70 |  |  |  |  |
|  |  |  |  |  |  | 8.12 | 8.38 | 1.30 | 0.59 |  |  |  |  |
|  |  |  |  |  |  | 8.47 | 9.36 | 3.80 | 0.55 |  |  |  |  |
