# Supplementary material for: Integrating 3D-printed sclero-corneal models into Rigid Gas Permeable contact lens training: A pilot study
Source: PLoS One. 2026 Sep 9;21(9):e0357490. doi: 10.1371/journal.pone.0357490 (PMC13557353; doi:10.1371/journal.pone.0357490)
Supplement: Supplementary file 2 [file pone.0357490.s002.docx]

**S2 File. Real Eyes Opinion questionnaire.** Questions of the Questionnaire after Real Eyes session.

1. In accordance with current data protection regulations, we inform you that the personal data collected in this survey will be treated confidentially and used exclusively for research purposes. Do you accept the processing of your personal data in accordance with this policy?

- Yes, I accept
- No, I do not accept

2. I declare that I haven been properly informed about the purpose and procedures of this survey, and I give my consent to participate voluntarily

- Yes, I consent
- No, I do not consent

3. Assigned number
4. Gender:

- Male
- Female
- Other

5. Age:
6. Rate the following aspects from 1 (least complex) to 5 (most complex):

- Choosing the initial lens
- Insertion and removal
- Fluorescein pattern assessment and parameter change decisions
- Dynamic lens evaluation
- Patient discomfort hindering evaluation

7. How much practice time do you estimate you spent cleaning the lenses to be able to place them on your partner?

- Very little
- Little
- Average
- A lot
- Too much

8. How much time do you estimate you spent on inserting and removing contact lenses?

- Very little
- Little
- Average
- A lot
- Too much

9. If you needed direct supervision from the professor when evaluating fluorescein patterns, did you have to wait because they were busy?

- I didn’t need supervision / I didn’t have to wait
- Sometimes I had to wait a bit
- I often had to wait a long time

10. Do you think it would be helpful to evaluate fluorescein patterns as a group with the initial guidance of the professor to develop more skill and discuss different fits?

- Not necessary
- Necessary

11. What discourages you most from wearing contact lenses and allowing your classmates to continue evaluating more fits? (Indicate for each: "Not at all", "Slightly", "Neutral", "A lot", "Too much")

- Discomfort during insertion and removal
- Discomfort while wearing RGP-CLs
- Improper lens disinfection
- Ill-fitting parameters causing eye irritation
