# Supplementary material for: Integrating 3D-printed sclero-corneal models into Rigid Gas Permeable contact lens training: A pilot study
Source: PLoS One. 2026 Sep 9;21(9):e0357490. doi: 10.1371/journal.pone.0357490 (PMC13557353; doi:10.1371/journal.pone.0357490)
Supplement: Supplementary file 3 [file pone.0357490.s003.docx]

**S3 File. Sclero-corneal surfaces Opinion questionnaire.** Questions of the Questionnaire after the session using Sclero-corneal surfaces.

1. Assigned number
2. Do you find similarities between evaluating fluorescein patterns on 3D printed surfaces and real eyes?

- No similarity
- Slight similarity
- Very similar and comparable

3. How useful did you find the simulation of fluorescein patterns on 3D printed surfaces?
    1 (Not useful at all) → 5 (Very useful)
4. To what extent do you feel you've acquired knowledge or skills you can apply in your future professional practice?

- Very little
- Little
- Average
- A lot
- Very much

5. Did you evaluate more fluorescein patterns using 3D printed surfaces or real-eye practice?

- More with artificial surfaces
- More with real eyes
- About the same

6. Do you think you learned more by not worrying about the pain or discomfort of inserting lenses?

- Yes
- No

7. Do you think discussing with classmates or the professor helped you better understand fluorescein patterns?

- Yes
- No

8. Do you think time usually spent disinfecting lenses in other sessions was well used when using artificial surfaces, given disinfection was unnecessary?

- Yes
- No

9. Do you think the new method provides a more dynamic and engaging learning experience?

- Yes
- No

10. Do you think 3D printing technology adds appeal to this type of practical activity?

- Yes
- No

11. Did the topographic map improved the learning process

- No
- Yes, it helped me better understand the corneal surface

12. Did practicing with 3D printed surfaces help you improve contact lens manipulation?

- Yes, I wasn’t afraid of harming a classmate
- No, I didn’t get to handle them as much as I wanted

13. Did you find it helpful to work with less common surfaces such as highly astigmatic corneas?

- Yes
- No

14. Do you think the time spent using 3D printed surfaces was appropriate?

- No, too much time
- No, too little time
- Yes, it was appropriate

15. Which method do you think is best suited for practicing each of the following? (Mark each as “Real Eye”, “Both”, or “Artificial Surface”)

- Insertion / Removal
- Lens handling
- Description of fluorescein patterns (tight/loose)
- Lens-cornea tear meniscus
- Need for design changes
- Knowledge of corneal surface parameters

16. When do you think it would be most useful to implement 3D printed surfaces practice?

- At the very beginning, even before using real eyes, to practice handling
- Alongside real eye practice to complement both methods
- After real eye practice to review concepts

17. Do you think it would be beneficial to implement 3D printed surfaces in the contact lens course moving forward?

- Yes
- No

18. Do you think the new method helped you better understand RGP-CLs fitting?

- No, it should be more complete
- Yes, it was very illustrative
