# Supplementary material for: Integrating 3D-printed sclero-corneal models into Rigid Gas Permeable contact lens training: A pilot study
Source: PLoS One. 2026 Sep 9;21(9):e0357490. doi: 10.1371/journal.pone.0357490 (PMC13557353; doi:10.1371/journal.pone.0357490)
Supplement: Supplementary file 4 [file pone.0357490.s004.docx]

**S4 File. Knowledge questionnaire.** Real eyes fluorescein pattern questions of the Knowledge questionnaire.

1. Which of the following best describes this fluorescein pattern?

- Excessive edge lift
- The contact lens is steeper than the cornea
- The mid-periphery is correctly fitted
- The central touch is ideal

2. Which of the following best describes this fluorescein pattern?

- Excessive edge lift
- The lens is correctly fitted in the central zone
- Excessively flat contact lens
- The lens shows excessive central clearance
